# Topoisomerase inhibitory activity of ethanolic extracts from botanicals Coptis chinensis and Salvia officinalis

**DOI:** 10.1099/jmm.0.002024

**Published:** 2025-06-03

**Authors:** Wade Ingersoll, Susan Trapp, Tiffany Turner, Guillermo Ruiz, Jeffrey Langland

**Affiliations:** 1 Sonoran University of Health Sciences, The Ric Scalzo Institute for Botanical Research, Tempe, AZ 85282, USA

**Keywords:** anticancer, antimicrobial, botanical, *Coptis chinensis*, *Salvia officinalis*, topoisomerase

## Abstract

**Introduction.** DNA topoisomerases are essential enzymes that allow cells to effectively manage the topological states of DNA. Due to the ubiquitous nature of their functions, topoisomerases have become promising treatment targets for various conditions, ranging from microbial infections to cancer.

**Hypothesis.** The botanicals, *Coptis chinensis* (Chinese goldthread) and *Salvia officinalis* (common sage), are herbs that boast a long history of traditional use for their effectiveness in treating a myriad of health concerns, including microbial infections and cancer, which could be associated with topoisomerase inhibitory activity.

**Aim.** This study sought to evaluate the antimicrobial and anticancer properties of these botanical extracts and determine if this activity was due to the presence of anti-topoisomerase activity.

**Methodology.** Using various bacterial genera, vaccinia virus, cancerous cell lines and topoisomerase activity assays, the activity of these extracts was evaluated.

**Results.** This study demonstrated that ethanolic extracts of these botanicals had potent anti-Gram-positive bacterial activity, antiviral activity and anticancer activity. Furthermore, this activity likely correlated with the ability of the extracts to inhibit topoisomerases II and IV and for *Salvia officinalis*, topoisomerase I.

**Conclusion.** These results support the potential therapeutic value of *C. chinensis* and *S. officinalis* for the treatment of health concerns.

## Introduction

DNA topoisomerases (topos) are essential enzymes that allow cells to effectively manage topological states of DNA. These enzymes introduce single- or double-strand breaks in DNA to regulate supercoiling, a phenomenon that occurs during DNA replication, transcription and other fundamental cellular processes [1,2]. Due to the ubiquitous nature of their functions, topos have become promising treatment targets for various conditions, ranging from microbial infections to cancer [3,4].

Quinolone antibiotics represent a critical class of drugs that inhibit topoisomerase activity in bacteria, namely topo II (DNA gyrase) and topo IV. These medications are widely used to treat a broad range of pathogenic infections such as prostatitis, tuberculosis and urinary tract infections [4,5]. In the 1970s, this class expanded following the development of fluoroquinolones, bringing to market broad-spectrum drugs, like ciprofloxacin and ofloxacin, that target both Gram-negative and Gram-positive bacteria [6]. The discovery of this class of antibiotics was a major contributing factor in the decline in mortality rates seen in the last century [7]. Quinolones have also been the subject of numerous *in vitro* cancer studies, exhibiting pro-apoptotic activity, halting cell cycle and initiating mitochondrial dysfunction [8].

Topos have also emerged as targets of interest in the research branch aimed towards antivirals. In a 2016 study, Freitas *et al*. employed siRNA knockdown techniques to investigate the function of a type II topo in African swine fever virus [9]. Their results showed an 89% decrease in viral-transcript load in knockdown-positive cells when compared to the control. Poxviruses have also been shown to encode a topo I, which is required for viral replication [10].

*Coptis chinensis* (Chinese goldthread) and *Salvia officinalis* (common sage) are herbs that boast a long history of traditional use for their effectiveness in treating a myriad of health concerns. Records dating back 2000 years support the use of *C. chinensis* for diabetes, owing to its modulation of blood sugar and insulin levels [11]. More recently, *C. chinensis* has demonstrated significant antiviral activity *in vitro*, reducing the replication of respiratory syncytial virus, one of the leading causes of infant mortality [12,13]. Similarly, the therapeutic benefit of *S. officinalis* is documented in traditional medicine practices throughout the globe, with a treatment range that includes minor ailments, such as sore throat, and more serious conditions, like neurodegenerative diseases [14,15]. Additionally, essential oil produced from *S. officinalis* has been shown to exhibit bactericidal capabilities against various strains of *Staphylococcus aureus* [16]. The long-standing evidence supporting the health benefits of *C. chinensis* and *S. officinalis* inspired their exploration in this study.

The present work aimed to investigate the potential antibacterial, antiviral and anticancer effects of *C. chinensis* and *S. officinalis*, with a focus on their mechanisms of action. It was found that botanical extracts from both herbs displayed potent anti-topo activity, likely responsible for observed antibacterial, antiviral and anticancer properties, and potentially other medicinal qualities described in the literature. Understanding the molecular basis of their activity could provide insights into the therapeutic benefit of other medicinal herbs and contribute to the development of new antimicrobial and anticancer agents.

## Methods

### Chemicals, reagents, bacteria, viruses and cell lines

Tryptic soy broth and tryptic soy agar were obtained from Hardy Diagnostics (Santa Monica, CA). Antibiotics, Mueller Hinton agar, reserpine and ethanol were obtained from Sigma-Aldrich Chemicals. Bacterial cultures of *Staphylococcus aureus* (ATCC 14775), *Streptococcus pyogenes* (ATCC 12344), *Bacillus cereus* (ATCC 10876), *Escherichia coli* (ATCC 11229), *Salmonella enteritidis* (ATCC 49223), *Shigella sonnei* (ATCC 29930) and *Pseudomonas aeruginosa* (ATCC 35554) were obtained from Hardy Diagnostics (Santa Monica, CA). HeLa (ATCC CCL-2), SiHa (ATCC HTB-35) and PANC-1 (ATCC CRL-1469) cells were maintained in Dulbecco’s Modified-Minimal Essential Medium (Cellgro) supplemented with 5% fetal bovine serum. All cells were incubated at 37 °C in the presence of 5% CO_2_. Vaccinia virus (Copenhagen strain), designated as vaccinia virus (VACV), was provided by Virogenetics.

### Preparation of plant material

Historically, *S. officinalis* leaf, *C. chinensis* root, and *Astragalus membranaceus* root are the portions of the plant typically used for medicinal purposes [12,15]. *S. officinalis* leaf and *A. membranaceus* root plant materials were obtained from Starwest Botanicals (Sacramento, CA) with high-performance TLC performed to verify purity and authenticity (Starwest Botanicals Certificate of Analysis for Product Numbers 209950-31 and 209140-31, respectively). *A. membranaceus* extracts were included in this study as a botanical control that, historically, has not been proposed to have direct antimicrobial activity or anti-topo activity. *C. chinensis* root was obtained from Mayway Corp/Plum Flower (Oakland, CA, Product Number 57915FP). All plant material was subsequently verified by qualified botanical specialists using herbal pharmacopoeia monographs and reference keys. A voucher specimen of all plant material was deposited in a repository at the Sonoran University of Health Sciences. For each plant, 5 g of dried leaves was ground to a fine powder using a high-speed blender, followed by resuspension in 50% ethanol at a ratio of 1:10 (dried plant material:extraction solution) and mixing by rotation at 60 r.p.m. at room temperature for 24 h. The botanical debris was removed by centrifugation (3,000 ***g*** for 10 min), and the supernatant sterilized by filtration through a 0.2 um filter. One millilitre of the final extract was dried to completion, and the concentration of non-volatile constituents was determined to be 35–45 mg ml^−1^. For assays using the extracts, the treatment doses ranged from 1 to 1,000 µg ml^−1^ based on the non-volatile constituents per millilitre. These doses are indicated in the figures and/or figure legends.

### Minimum bactericidal concentration assay

For bacterial inhibition studies, minimum bactericidal concentrations (MBC) were determined [17]. MICs were not measured due to the turbidity of the broth cultures, which occurred following the addition of the botanical extracts, making a reliable determination of MIC values difficult. For MBC determination and growth studies, 18 h broth cultures (5×10⁸ c.f.u. ml^−1^) were diluted into broth media [1:1,000 dilution; tryptic soy broth (TSB)] followed by the addition of indicated concentrations of each botanical extract or null control. The cultures were incubated at 37 °C with aeration (by continuous rotation) for 24 h. The MBCs were determined by serial dilution of the broth cultures and plating onto tryptic soy agar plates and incubation for 24 h at 37 °C. The MBC was identified by determining the lowest concentration of the treatment that reduced the viability of the bacteria by ≥99.9%. For the botanical extracts, the MBC was listed as the total concentration of non-volatile constituents present in the extract since the active constituents have not been isolated or identified. Assays were done with comparable doses of vehicle alone (50% EtOH), which demonstrated no inhibitory effects on bacterial growth (data not shown). For studies using reserpine, 10 µg ml^−1^ reserpine was added concomitantly with the botanical extract. Control experiments indicated that the MIC and MBC of reserpine alone were greater than 50 µg ml^−1^ (data not shown).

Antibiotic susceptibility testing was performed using the Kirby–Bauer disc diffusion method according to the Clinical and Laboratory Standards Institute (CLSI: M100-S22) guidelines [17]. Bacterial suspensions were prepared by transferring 3–5 pure colonies into the nutrient broth and adjusted to 0.5 McFarland standards. A sterile cotton swab was then dipped into the suspension and swabbed on the surface of the Mueller–Hinton agar plate. Standard antibiotic discs were placed aseptically, and the inoculated Mueller–Hinton agar plates were incubated at 37 °C for 16–18 h (penicillin 10 µg, ciprofloxacin 5 µg and gentamicin 10 µg). The diameters of the zones of complete inhibition were measured using callipers in mm.

### Bacterial botanical resistance development

In order to help determine the antibacterial mechanism of action of the botanical extracts, bacterial strains resistant to the extracts were developed. *Staphylococcus aureus* (ATCC 14775) was used as a model organism to study the antibacterial activity of the botanicals described in this study. A *Staphylococcus aureus* strain resistant to the botanical extracts was previously described and used in these studies [17]. As previously described, *Staphylococcus aureus* (ATCC 14775) cultures (1×10^6^ c.f.u. ml^−1^) in TSB were treated with a 75% MBC dose of the botanical extract. The cultures were incubated at 37 °C with continuous aeration. Every 24 h, the bacterial culture was transferred to five different vials of fresh TSB media containing increasing amounts of the antimicrobial. The vial that demonstrated bacterial growth at the highest dose of antimicrobial was selected to continue the selection process. This process was repeated for a total of 15 days.

### Topoisomerase inhibitor assay

Topoisomerase II (Gyrase), topoisomerase I (human) and topoisomerase (*E. coli*) IV drug screening kits were obtained from TopoGen (Buena Vista, CO). Assays were done following the manufacturer’s recommended procedures. Briefly, 0.25 µg pHOT plasmid DNA (relaxed plasmid for topoisomerase II or supercoiled plasmid for topoisomerase I and IV) was incubated in the presence or absence of the topoisomerase enzyme and the indicated concentrations of ciprofloxacin or botanical extract for 15 min at 37 °C. The reactions were terminated by the addition of sodium dodecyl sulphate, and products were analysed on a 1% agarose gel containing 0.5 ug ml^−1^ ethidium bromide.

### VACV plaque assay

HeLa cells were infected with 100 pfu of VACV for 60 min, washed two times with media and treated with the indicated concentrations of each botanical extract. Cells were incubated at 37 °C in the presence of 5% CO_2_ for 48 h. VACV plaques were visualized by crystal violet staining [18].

### Cell viability assay

SiHa or PANC-1 cells were treated with the indicated concentrations of the botanical extracts for 24 h. Cell viability was assessed using the standard colourimetric MTS assay (Abcam MTS Assay Kit ab19701) following the manufacturer’s recommended protocol.

### Statistical analysis

Statistical analysis was performed using a paired t-test. Samples with statistically significant differences are indicated with the calculated *P*-value or asterisks (*P=0.01–0.05; **P=0.001–0.01).

## Results

**Fig. 1.**
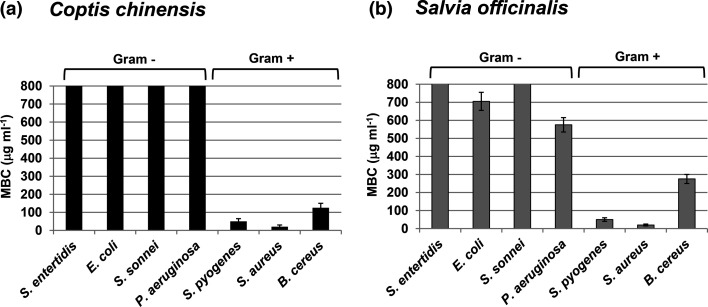
Antibacterial activity of *C. chinensis* and *S. officinalis* extracts. Gram-negative and Gram-positive bacteria were treated with ethanolic extracts of *C. chinensis* (**a**) and *S. officinalis* (**b**). MBC was calculated as microgram of non-volatile constituents per millilitre of extract. MBC values of 800 µg ml^−1^ indicate no antibiotic activity. Error bars denote the standard deviation from three separate experiments.

To evaluate the antibacterial properties of *C. chinensis* and *S. officinalis*, ethanolic extracts of each botanical were prepared and incubated with Gram-positive (*Streptococcus pyogenes*, *Staphylococcus aureus* and *B. cereus*) and Gram-negative bacteria (*Salmonella enteritidis*, *E. coli*, *Shigella sonnei* and *P. aeruginosa*). The extracts were administered in a dose-curve fashion to identify the MBC, which was measured in microgram of non-volatile constituents per millilitre of extract (µg ml^−1^). As shown in Fig. 1a and b, both botanical extracts preferentially demonstrated antibacterial activity against the Gram-positive bacteria compared to the Gram-negative bacteria. The MBC values for *C. chinensis* (Fig. 1a) against the Gram-positive strains were *Streptococcus pyogenes*, 50±15 µg ml^−1^; *Staphylococcus aureus,* 20±10 µg ml^−1^; and *B. cereus*, 125±25 µg ml^−1^, whereas for the Gram-negative bacteria, no inhibition was observed over the concentrations tested (up to 800 µg ml^−1^). Similarly, for *S. officinalis*, the extracts had Gram-positive MBC values of *Streptococcus pyogenes*, 50±10 µg ml^−1^; *Staphylococcus aureus*, 20±5 µg ml^−1^; and *B. cereus*, 275±25 µg ml^−1^, whereas only minor activity was observed with the Gram-negative (*E. coli*, 705±50 µg ml^−1^, and *P. aeruginosa*, 575±40 µg ml^−1^). Assays done with comparable doses of vehicle alone (50% EtOH) had no inhibitory effects on bacterial growth (data not shown). These results may suggest that the active antibacterial constituent(s) present in these botanical extracts are impermeable to the outer lipopolysaccharide layer of Gram-negative bacteria, allowing uptake only into Gram-positive bacterial species.

**Fig. 2.**
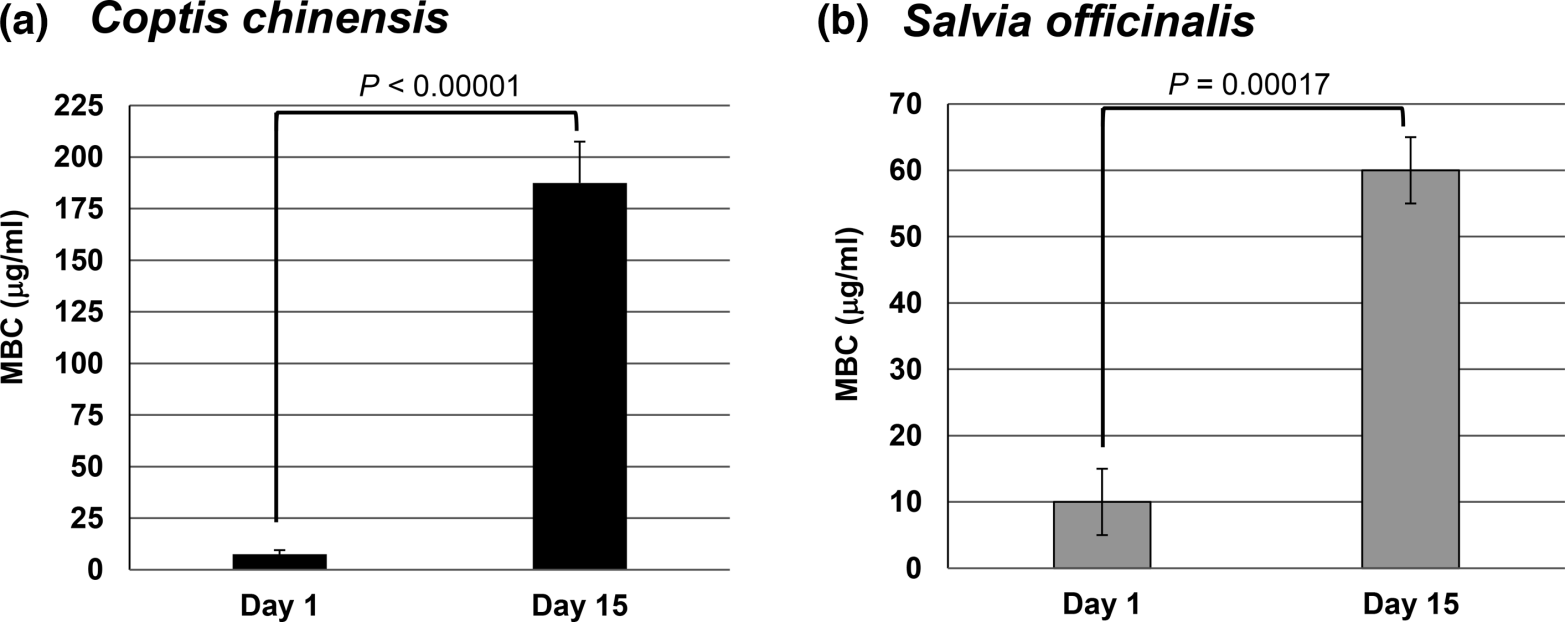
Resistance development of *Staphylococcus aureus* to botanical extracts. Cultures of *Staphylococcus aureus* were exposed to sub-inhibitory doses of *C. chinensis* (**a**) and *S. officinalis* (**b**) extracts for a period of 15 days to select for the development of resistance. MBC was calculated on days 1 and 15 to compare susceptibility before and after the selection process. MBC was recorded as µg of non-volatile constituents per millilitre of extract. Error bars denote the standard deviation from three separate experiments. Statistical analysis was done using a paired t-test and the *P*-value indicated.

To begin to assess the potential mechanism of action of these botanical extracts, resistant bacterial strains were developed. As previously described, *Staphylococcus aureus* was grown in the presence of sub-inhibitory doses of each botanical extract over a 15-day period [17]. MBC was measured on days 1 and 15 to compare susceptibility. The *C. chinensis* resistant strain exhibited a 25-fold increase (*P*<0.00001) in MBC from day 1 (7.5±2 µg ml^−1^) to day 15 (187.5±20 µg ml^−1^) (Fig. 2a), and the *S. officinalis* resistant strain displayed similar effects, although less pronounced, with MBC increasing sixfold (*P*<0.00017) from day 1 (10±5 µg ml^−1^) to day 15 (60±5 µg ml^−1^) (Fig. 2b). The increase in MBC indicates a higher concentration was necessary to achieve the same effect as day 1, confirming the establishment of resistance. Interestingly, *C. chinensis* induced a greater increase in MBC, suggesting that *Staphylococcus aureus* developed resistance more effectively toward it than *S. officinalis*.

**Fig. 3.**
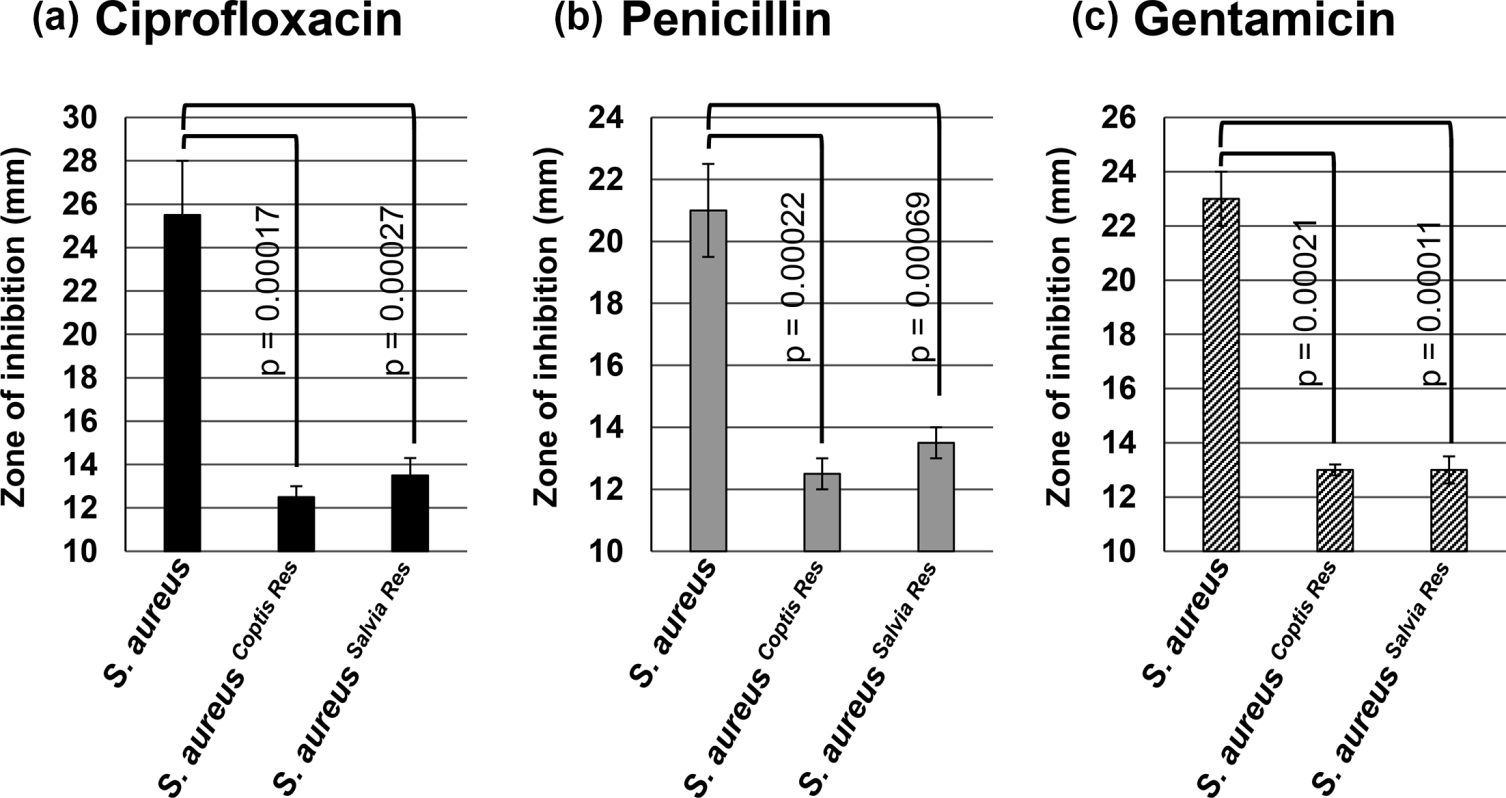
Antibiotic sensitivity of botanical-resistant strains of *Staphylococcus aureus*. The botanical extract-resistant strains (*Staphylococcus aureus ^Coptis Res^* and *Staphylococcus aureus ^Salvia Res^*) and a non-resistant strain of *Staphylococcus aureus* were treated with ciprofloxacin (**a**), penicillin (**b**) and gentamicin (**c**) in a disc diffusion assay. The treatment doses included penicillin at 10 µg, ciprofloxacin at 5 µg and gentamicin at 10 µg. The diameter of the ZOI was recorded. Error bars denote the standard deviation from three separate experiments. Statistical analysis was done using a paired t-test and the *P*-value indicated.

Bacteria develop resistance to antimicrobial compounds through a variety of mechanisms, including efflux pumps, which actively expel toxic material from the cell [19,20]. A key characteristic of efflux pumps is their ability to confer resistance to a range of different compounds, beyond the one that initially induced resistance (cross-tolerance) [21]. The *Staphylococcus aureus* sensitive and botanical extract-resistant strains (shown in Fig. 2) were treated with various pharmaceutical antibiotics to determine any cross-tolerance. As expected, when the zone of inhibition (ZOI) was determined for each antibiotic against the non-resistant strain of *Staphylococcus aureus*, the bacteria was found to be sensitive to all the antibiotics tested (ciprofloxacin, 25.5±2.5 mm; penicillin, 21±1.5 mm; gentamicin, 23±1 mm) (Fig. 3). When the *C. chinensis*-resistant (*Staphylococcus aureus ^Coptis Res^*) and *S. officinalis*-resistant (*Staphylococcus aureus ^Salvia Res^*) strains were treated with ciprofloxacin, penicillin and gentamicin in the disc diffusion assay, the resistant strains demonstrated significantly smaller ZOIs compared to the non-resistant strain (Fig. 3). For *Staphylococcus aureus ^Coptis Res^*, the ZOIs for ciprofloxacin, penicillin and gentamicin were 12.5±0.5 mm, 12.5±0.5 mm and 13±0.2 mm, respectively, and for *Staphylococcus aureus ^Salvia Res^*, the ZOI values were 13.5±0.8 mm for ciprofloxacin, 13.5±0.5 mm for penicillin and 13±0.5 mm for gentamicin (Fig. 3a–c). The lower ZOI values indicate that the antibiotics were less effective against the extract-resistant strains, demonstrating their cross-tolerance to the drugs. This cross-tolerance is consistent with efflux pump activity potentially being responsible for the resistance mechanism displayed by *Staphylococcus aureus ^Coptis Res^* and *Staphylococcus aureus ^Salvia Res^*.

**Fig. 4.**
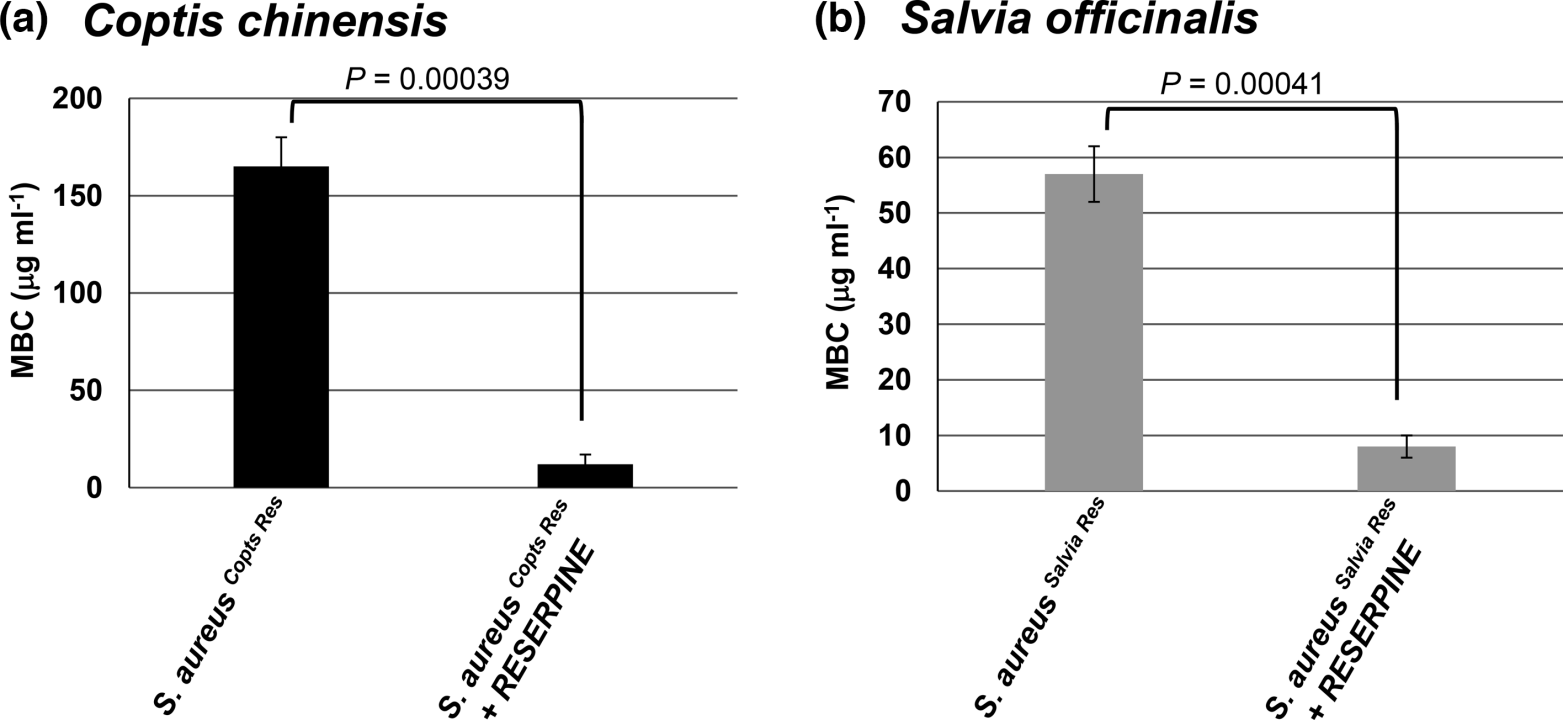
Effect of reserpine on botanical-resistant strains of *Staphylococcus aureus*. The botanical extract-resistant strains (*Staphylococcus aureus ^Coptis Res^* and *Staphylococcus aureus ^Salvia Res^*) were treated with *C. chinensis* (**a**) and *S. officinalis* (**b**) extracts with and without reserpine (an efflux pump inhibitor). The MBC was calculated for each condition. MBC was recorded as µg of non-volatile constituents per millilitre of extract. Error bars denote the standard deviation from three separate experiments. Statistical analysis was done using a paired t-test and the *P*-value indicated.

To support the involvement of an efflux pump in the resistance of the extract-resistant strains, the extract-resistant strains were treated with their respective botanical extract in the presence of an efflux pump inhibitor, reserpine [22], and the MBC was measured. *Staphylococcus aureus ^Coptis Res^* treated with the *C. chinensis* extract and reserpine exhibited an approximate 13-fold decrease (*P*=0.00039) in MBC compared to treatment without reserpine (*C. chinensis*, 165±15 µg ml^−1^; *C. chinensis*+reserpine; 12±5 µg ml^−1^) (Fig. 4a). The MBC of the extract with reserpine was similar to that of the pre-resistance value (7.5±2 µg ml^−1^) (Fig. 2a). Similarly, *S. officinalis* with reserpine showed an approximate sevenfold decrease (*P*=0.00041) in MBC compared to the extract alone (*S. officinalis*, 57±5 µg ml^−1^; *S. officinalis*+reserpine; 8±2 µg ml^−1^) (Fig. 4b), with MBC nearly matching the pre-resistance value (10±5 µg ml^−1^) (Fig. 2b). The ability of reserpine to restore the antimicrobial activity of the extract supports that an efflux pump contributes to the resistance in *Staphylococcus aureus ^Coptis Res^* and *Staphylococcus aureus ^Salvia Res^*.

**Fig. 5.**
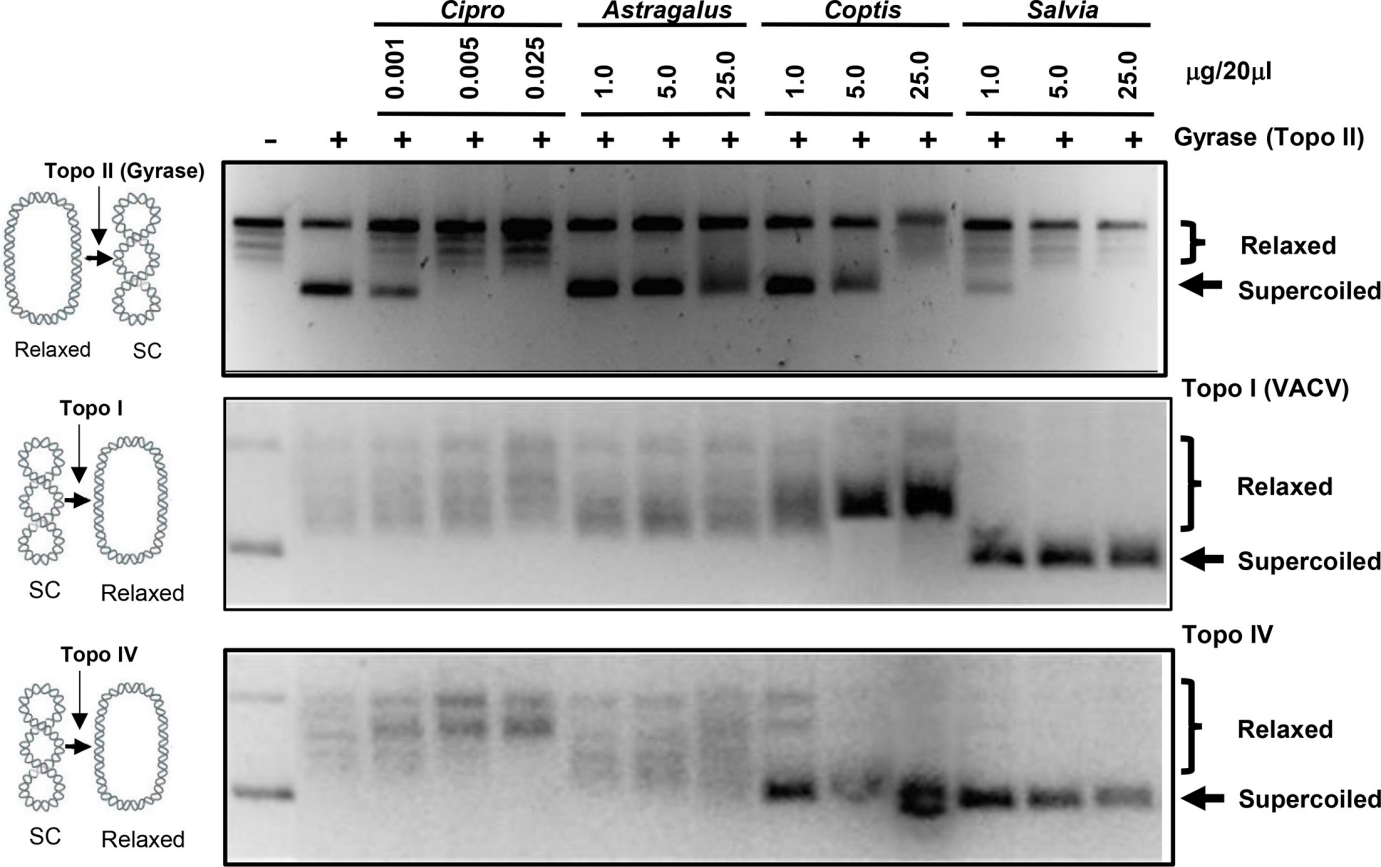
Topoisomerase inhibition of botanical extracts. The inhibitory activity of *C. chinensis, S. officinalis, A. membranaceus* extracts and ciprofloxacin against DNA gyrase (topo II) (top), topo I (middle) and topo IV (bottom). The first two lanes represent the plasmid in the absence or presence of the topo enzyme. Subsequent lanes represent the plasmid with the topo enzyme and indicate sample/dose. Arrows indicate the positions of the supercoiled and relaxed DNA bands.

The activity of efflux pumps often increases following exposure to fluoroquinolones [23]. Based on our results from Figs3  4, we proposed that the *Coptis* and *Saliva* botanical extracts may contain a similar topo inhibitor. To test this, the extracts were tested using commercial kits testing for activity against topo I, topo II (DNA gyrase) and topo IV. In these assays, the ability of the extracts to inhibit the conversion of a relaxed to supercoiled plasmid in the presence of topo II or to inhibit the conversion of a supercoiled to relaxed plasmid in the presence of topo I or topo IV was determined. Ciprofloxacin is a known inhibitor of topo II [24] and served as a positive control where the addition of ciprofloxacin inhibited DNA gyrase (topo II) at 0.005 and 0.025 µg/20 µl, but did not inhibit topo I or IV (Fig. 5). For the botanical extracts, the *S. officinalis* extract inhibited topo I and IV at all treatment doses (Fig. 5, middle and bottom) and DNA gyrase (topo II) at the two highest doses (5 and 25 µg/20 µl) (Fig. 5, top). The *C. chinensis* extract inhibited topo IV at all doses (Fig. 5, bottom), DNA gyrase (topo II) at the highest dose (Fig. 5, top), but did not inhibit topo I (Fig. 5, middle). *A. membranaceus* is a medicinal herb known to lack antibiotic properties and serves as a negative ‘botanical’ control to determine the specificity of the *C. chinensis* and *S. officinalis* extract effects. As shown in Fig. 5, the *A. membranaceus* extract did not exhibit substantial inhibitory activity for topo II, topo I or topo IV. The results for the *C. chinensis* and *S. officinalis* extracts to inhibit DNA gyrase (topo II) support the possible antibacterial mechanism of action observed in Fig. 1.

**Fig. 6.**
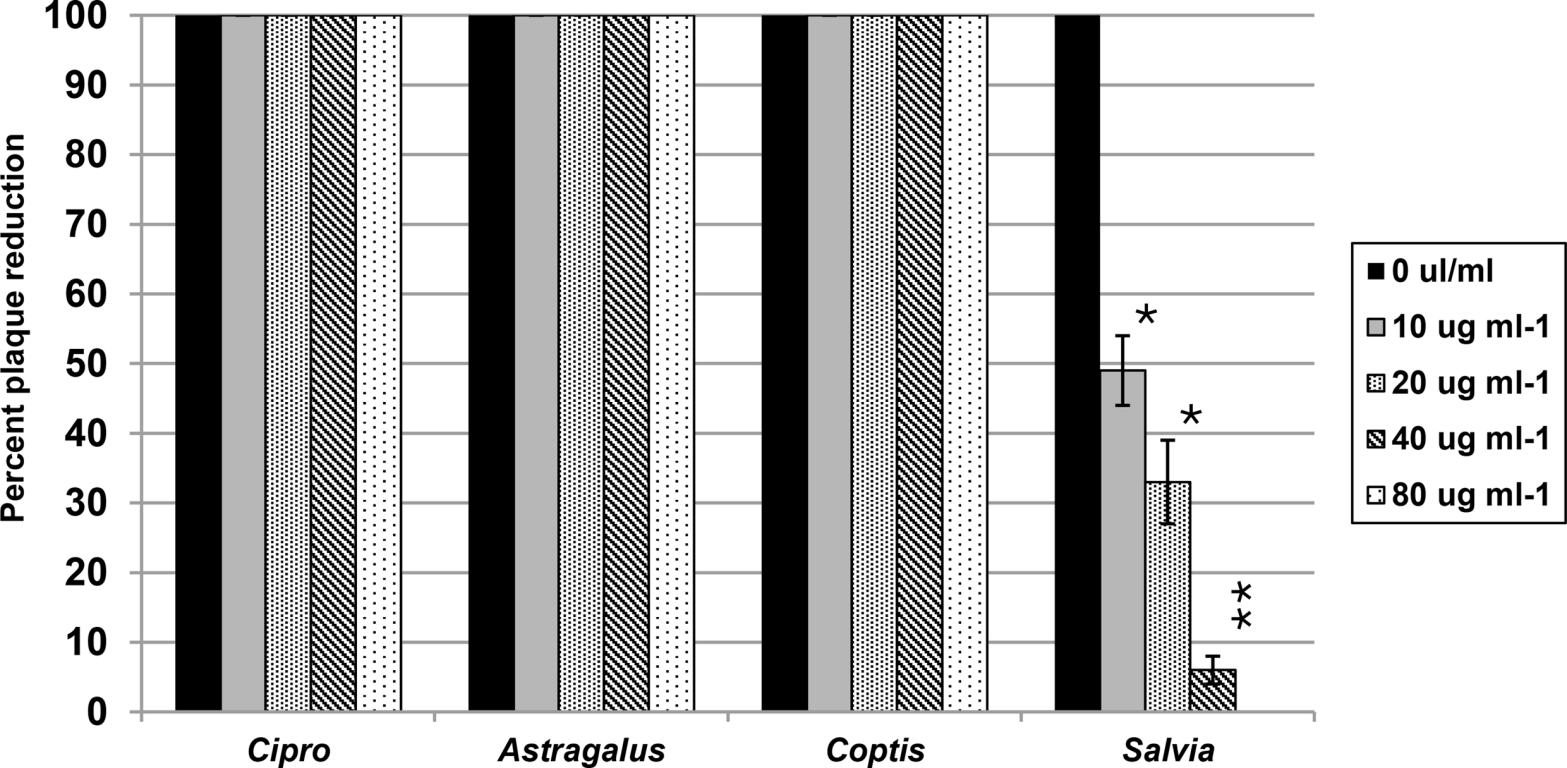
Anti-VACV activity of the botanical extracts. HeLa cells were infected with 100 p.f.u. of VACV and treated with *C. chinensis, S. officinalis, A. membranaceus* and ciprofloxacin. Viral inhibition was quantified by counting plaques after treatment. Each cluster of bars represents a treatment group, with each bar within the cluster indicating a different treatment dose. Error bars denote the standard deviation from three separate experiments. Statistical analysis was done comparing each treatment to the 0 µg ml^−1^ dose within the same treatment group. Statistically significant differences are indicated with an asterisk (*P=0.01–0.05; **P=0.001–0.01).

Since topo I is required by poxviruses for viral replication, the ability of the *S. officinalis* extract to inhibit the replication of VACV was evaluated. *C. chinensis* was included as a negative control since it was shown not to have anti-topo I activity. A plaque reduction assay was performed with VACV in the presence of the botanical extracts. As shown in Fig. 6, the *S. officinalis* extract inhibited replication in a dose-dependent manner with an IC_50_ of ~10 µg ml^−1^, while *C. chinensis* had no effect. Neither of the controls, ciprofloxacin or the *A. membranaceus* extract, inhibited viral replication. The results with the *S. officinalis* extract agree with the results from Fig. 5, where the *S. officinalis* extract was shown to inhibit topo I, which is required for VACV replication.

**Fig. 7.**
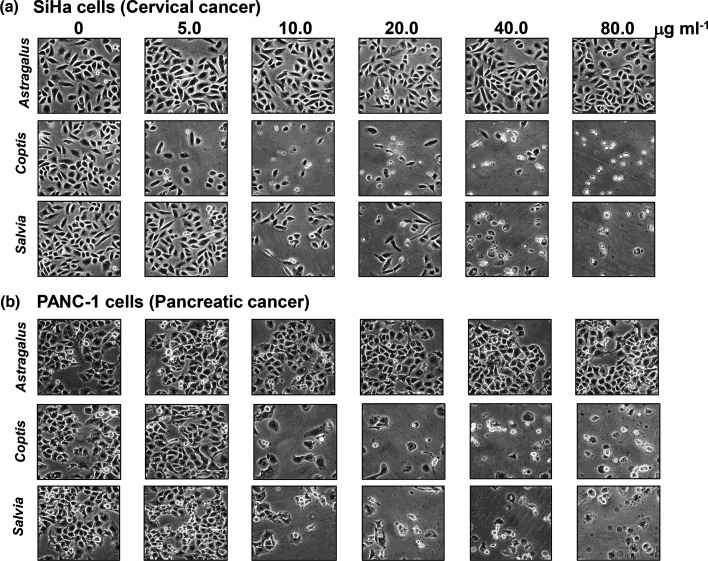
Cancer cell morphology after treatment with botanical extracts. SiHa (**a**) and PANC-1 (**b**) cells were treated with *C. chinensis, S. officinalis* and *A. membranaceus* extracts. After 24 h, photographs were taken under the microscope (100×) to visually observe the cell morphology. Treatment doses are listed at the top of the figure in µg ml^−1^.

**Fig. 8.**
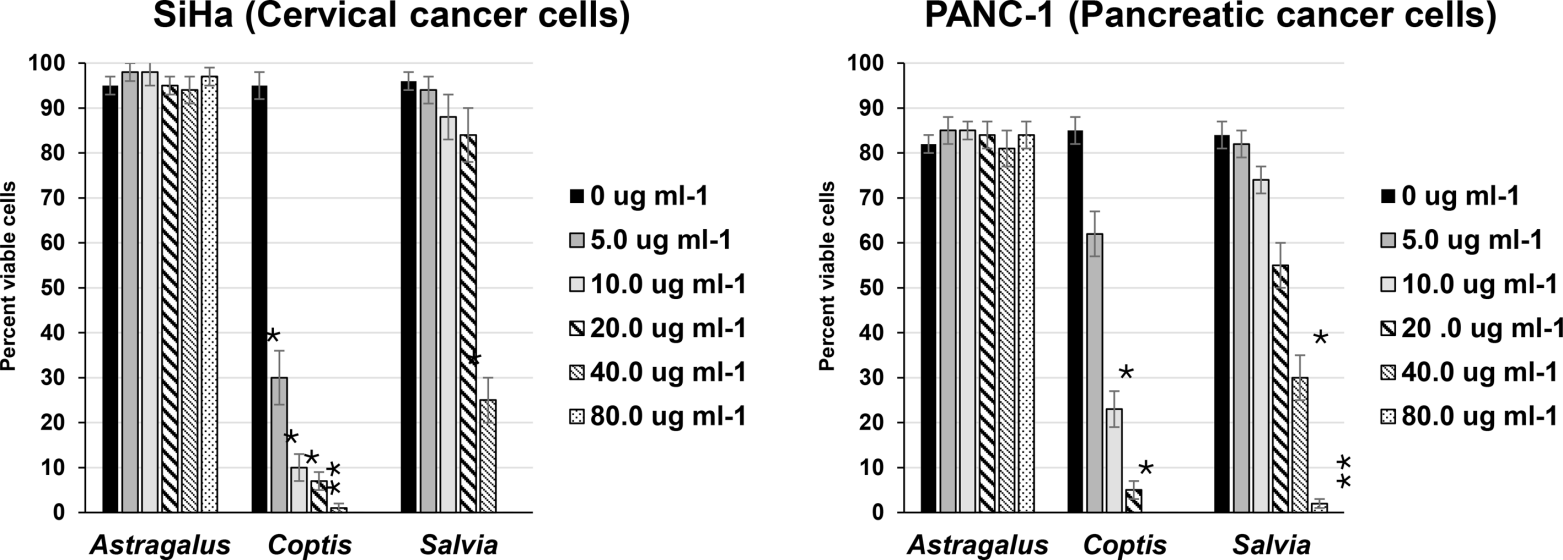
Cancer cell viability after treatment with botanical extracts. SiHa and PANC-1 cells were treated with *C. chinensis, S. officinalis* and *A. membranaceus* extracts. After 24 h, cell viability was assessed using a standard MTS assay. Each cluster of bars represents a treatment group, with each bar within the cluster indicating a different treatment dose. Error bars denote the standard deviation from three separate experiments. Statistical analysis was done comparing each treatment to the 0 µg ml^−1^ dose within the same treatment group. Statistically significant differences are indicated with an asterisk (*P=0.01–0.05; **P=0.001–0.01).

Inhibitors of topoisomerases, especially topo II, are proven therapeutic targets against cancer. Based on this, the botanical extracts were investigated for their effects on cancer cells. SiHa and PANC-1 cell lines, derived from cervical and pancreatic cancer, respectively [25], were treated with increasing doses of the *C. chinensis* and *S. officinalis* extracts (0, 5, 10, 20, 40 and 80 µg ml^−1^) and analysed qualitatively by microscopy (Fig. 7) and quantitatively by MTS assay (Fig. 8). As shown, both botanical extracts had significant cancer cell cytotoxicity at 5–10 µg ml^−1^ for the *C. chinensis* extract and 40–80 µg ml^−1^ for the *S. officinalis* extract. The *A. membranaceus* extract, which had no anti-topo activity from Fig. 5, has no measurable cytotoxic effects on the cancer cell lines and was again used as a negative ‘botanical’ control. These cancer cell cytotoxicity results for the *C. chinensis* and *S. officinalis* extracts are in agreement with Fig. 5, where both extracts demonstrated anti-topo activity, including anti-topo II activity.

## Discussion

The field of botanical medicine has garnered significant interest in recent years, owing to evidence of therapeutic efficacy and reduced risk of off-target effects [26]. Within this field, *C. chinensis* (Chinese goldthread) and *S. officinalis* (common sage) play an integral role in traditional medicine practices for their ability to treat a variety of diseases, ranging from cancer to microbial infections [27,29]. Despite their extensive use, the mechanisms by which *C. chinensis* and *S. officinalis* confer their therapeutic benefits are relatively understudied. Much of the existing research explains their effects by merely detailing potential bioactive constituents contained within each plant, neglecting to offer insights into the mechanisms through which these effects occur. For instance, the antimicrobial activity of *C. chinensis* is often attributed to a compound called berberine that is highly abundant in the plant, but comprehensive discussions of its molecular interactions are limited [30,32]. Similarly, research on *S. officinalis* focuses on the structure of compounds contained within the plant, often comparing them to known biologically active molecules, but mechanistic explanations for their effects are lacking [33]. This study investigated the antimicrobial and anticancer properties of *C. chinensis* and *S. officinalis* extracts while further elucidating the potential mechanisms of action. The results highlight two key findings: (1) both *C. chinensis* and *S. officinalis* possess antibacterial and anticancer properties, while the *S. officinalis* extract also had antiviral activity, and (2) both extracts exhibited inhibitory activity towards topo II and IV, with the *S. officinalis* extract also inhibiting topo I. These results are summarized in Table 1.

**Table 1.** Summary of the activity of the botanical extracts. The table summarizes the inhibitory activity of *C. chinensis, S. officinalis, A. membranaceus* extracts and ciprofloxacin against topo I, topo II, topo IV, *Staphylococcus aureus*, VACV and cancer cells. A single + indicates moderate inhibitory activity, while a double ++ indicates high activity. A single – indicates no inhibition. nd, not determined

	TOPO II inhibition	Topo I inhibition	Topo IV inhibition	*Staphylococcus aureus* inhibition	VACV inhibition	Cancer cell toxicity
**Cipro**	**++**	−	−	**+**	−	**nd**
***Astragalus***	−	−	−	−	−	−
***Coptis***	**+**	−	**+**	**+**	−	**+**
***Salvia***	**++**	**++**	**++**	**+**	**+**	**+**

A common feature of all topos is their ability to transform the topological states of DNA. Antibiotic compounds that possess more than one target in separate but related pathways can be described as multitargeted. Most of the antibiotics that have boasted long-term success fall into this category [34]. The previously described data highlight the ability of *C. chinensis* and *S. officinalis* extracts to inhibit several topoisomerase enzymes, indicating their multitarget nature and, thus, suggesting their potential for long-term success as antimicrobial therapeutics. Unfortunately, the antibacterial properties of the *C. chinensis* and *S. officinalis* extracts are primarily limited to Gram-positive organisms. Not to be underscored, Gram-positive bacteria include many highly pathogenic microbes supporting the value of these botanical extracts. However, the data may suggest that if the antibacterial properties of *C. chinensis* and *S. officinalis* are associated with a topo inhibitor, this constituent is likely unable to be taken up through the Gram-negative lipopolysaccharide membrane. First-generation quinolone drugs had similar Gram-positive specific limitations. The newer second generation of compounds, such as ciprofloxacin, display greater broad-spectrum activity against both Gram-positive and Gram-negative bacteria. These newer molecules introduced a fluorine at position C6 and a major ring substituent (piperazine or methyl-piperazine) at C7, leading to the term ‘fluoroquinolones’ [35].

Topos are crucial for the rapid division and proliferation of cancer cells, making them attractive treatment targets for cancer [36]. Current treatments include camptothecin and doxorubicin, which target type 1 (topo I, III and IV) and type 2 (topo II, IV and VI) topoisomerases, respectively [37,38]. While these drugs are effective, they bring significant side effects. As shown, the extracts of *C. chinensis* and *S. officinalis* showed that both botanical extracts demonstrate significant anticancer properties (Figs.7^8^) and act on both type 1 (topo I) and 2 (topo II (for *S. officinalis*) and IV) topoisomerases.

Vaccinia virus (VACV) is one of the few viruses that encode a type 1 topoisomerase [10]. The enzyme plays a similar role to that of DNA gyrase and topo IV in bacteria, providing relief of DNA supercoiling. Given the anti-topo I activity of the *S. officinalis* extract, the observed anti-VACV effect of the *S. officinalis* extract was expected and observed. In contrast, *C. chinensis*, which did not inhibit topo I, did not show significant effects on VACV replication. The consistency between the expected and actual results highlights the reliability of this study’s findings and validates the antiviral properties of *S. officinalis*.

Throughout this study, *A. membranaceus* was used as a negative ‘botanical’ control to ensure that the observed effects were specific to the *C. chinensis* and *S. officinalis* extracts and not simply a result of an ethanolic botanical extract in general. The anti-topo, antiviral and anticancer experiments all included the *A. membranaceus* extract, where no substantial activity was observed, thereby confirming the specificity of *C. chinensis* and *S. officinalis*.

One significant limitation of this study is the lack of direct evidence linking the antibacterial, antiviral and anticancer effects of the *C. chinensis* and *S. officinalis* extracts to the direct inhibition of topo activity. Addressing this would require isolation of the compound and showing its ability to inactivate topo in conjunction with the biological activity. Since these compounds have not yet been identified or isolated, future research will be conducted with this objective.

The results presented demonstrate that *C. chinensis* and *S. officinalis* extracts contain antibacterial, anticancer and antiviral (for *S. officinalis*) properties likely associated with topoisomerase inhibition. Future studies will focus on the isolation of the active constituents within each botanical. It remains unclear whether the extracts contain multiple active compounds, each targeting different topoisomerases, or if a single multi-targeted compound is acting on different topos. Whichever the case, this offers an opportunity to potentially identify novel topoisomerase inhibitors and provide deeper insights into the molecular mechanisms underlying the activity of *C. chinensis* and *S. officinalis*.

## Abbreviations

MBC: minimum bactericidal concentrations
topos: topoisomerases
TSB: tryptic soy broth
VACV: vaccinia virus
ZOI: zone of inhibition
